# Upregulation of Oxidative Stress Related Genes in a Chronic Kidney Disease Attributed to Specific Geographical Locations of Sri Lanka

**DOI:** 10.1155/2016/7546265

**Published:** 2016-11-16

**Authors:** Saravanabavan Sayanthooran, Dhammika N. Magana-Arachchi, Lishanthe Gunerathne, Tilak D. J. Abeysekera, Suneth S. Sooriyapathirana

**Affiliations:** ^1^Cell Biology, National Institute of Fundamental Studies, Kandy, Sri Lanka; ^2^Renal Research Centre, District Hospital, Girandurukotte, Sri Lanka; ^3^Department of Pharmacology, Faculty of Medicine, University of Peradeniya, Peradeniya, Sri Lanka; ^4^Department of Molecular Biology and Biotechnology, Faculty of Science, University of Peradeniya, Peradeniya, Sri Lanka

## Abstract

*Objective.* To infer the influence of internal and external oxidative stress in chronic kidney disease patients of unknown etiology (CKDu) in Sri Lanka, by analyzing expression of genes related directly or indirectly to oxidative stress: glutamate-cysteine ligase catalytic subunit (GCLC), glutathione S-transferase mu 1 (GSTM1), glucose-6-phosphate dehydrogenase (G6PD), fibroblast growth factor-23 (FGF23), and NLR family pyrin domain containing 3 (NLRP3).* Methods.* Reverse transcription quantitative polymerase chain reaction (RT-qPCR) was carried out for the selected populations: CKDu patients (*n* = 43), chronic kidney disease patients (CKD; *n* = 14), healthy individuals from a CKDu endemic area (GHI; *n* = 9), and nonendemic area (KHI; *n* = 16). Fold changes were quantified relative to KHI.* Results.* GCLC had greater than threefold upregulation in all three study groups, with a maximum of 7.27-fold upregulation in GHI (*p* = 0.000). GSTM1 was not expressed in 25.6% of CKDu and 42.9% of CKD patients, but CKDu patients expressing GSTM1 showed upregulation of 2.60-fold (*p* < 0.05). Upregulation of FGF23 and NLRP3 genes in CKD and CKDu was observed (*p* < 0.01), with greater fold changes in CKD.* Conclusion.* Results suggest higher influence of external sources of oxidative stress in CKDu, possibly owing to environmental conditions.

## 1. Introduction

Chronic kidney disease (CKD) is on the increase globally with 10 percent of the worldwide population being affected by CKD and millions dying each year due to lack of access to treatment and to end-stage renal disease [1]. This global increase of CKD is majorly secondary to an increase in diabetes, infections, and cardiovascular and autoimmune disorders [2].

In developed countries, generally the risk factors include type 2 diabetes, hypertension, obesity, and ageing. Glomerular nephritis and interstitial nephritis are the major causes of CKD in developing countries and point towards microbial infections and environmental toxins as risk factors [3]. Diabetes and hypertension are also risk factors in the developing world with diabetes causing 9.1 to 29.9 percent of the cases of ESRD and hypertension causing 13 to 21 percent of the cases [3].

In Sri Lanka, between 1990 and 2007, hospital admissions due to all diseases of the genitourinary system doubled with hospital deaths due to such diseases rising from 2.6 to 9.1 per 100,000 people [4]. CKD has gained a lot of importance in Sri Lanka due its rise in alarming rates in many regions of the country in which its cause is yet unknown/uncertain. The Sri Lankan government and World Health Organization (WHO) hence have given it the name chronic kidney disease of unknown etiology (CKDu). It is estimated that around 400,000 people in Sri Lanka's north central region may be affected by the disease [5].

This peculiar form of CKD is not specific to Sri Lanka, and similar cases have been recorded across three continents around the world [6], mostly in agricultural communities, of Latin America [7, 8], Egypt [9], and India [10, 11].

Reactive oxygen species (ROS) and nitrogen species (RNS) are essential for maintaining the healthy physiological state of an individual; however, when it exceeds the necessary amounts, it leads to oxidative stress, which is the pathophysiological mechanism for many chronic diseases including CKD [12]. The expressions of the oxidative stress related genes alter depending on both the internal and external oxidative stress. We therefore hypothesize that studying the differential expression of genes related to oxidative stress response in CKD, CKDu, and healthy individuals of a CKDu-affected endemic region and comparing them to healthy individuals of an unaffected region would reveal the contribution of oxidative stress from the environment. As majority of the hypotheses for causes of CKDu are environmental related, studying the expressions of oxidative stress related genes in these populations of different geographical areas would result in better understanding the influence of the environment in this disease.

The oxidative stress related genes selected for the study include the glutamine cysteine ligase C subunit (GCLC); glutathione S transferase mu 1 (GSTM1); and the glucose 6 phosphate dehydrogenase (G6PD) genes. Two of these genes, GCLC and GSTM1, are related to the glutathione (GSH) protein. The G6PD gene was selected as it plays a role in protection against reactive oxygen species by the production of NADPH which is required for the generation of reduced GSH, as well as due to its deficiency being linked to CKDu in another study [13]. Polymorphisms of these genes, although not in combination, have been seen in cases of CKDu and end-stage renal disease (ESRD) in Mexican [14], Indian [15], and Sri Lankan populations [13].

ROS is however generated in CKD by several internal processes including hyperactivation of NADPH oxidase, increased production of oxidative stress markers, and the release of uremic toxins [16]. We therefore analyzed the expression pattern of genes reacting to internal sources of oxidative stress production to better understand the environmental versus physiological sources of oxidative stress in the study groups.

The increased oxidative stress in chronic kidney disease is in part attributed to the dysfunctional mitochondria which cause an increased electron leakage from the respiratory chain during oxidative phosphorylation with a consequent generation of ROS [16]. NLR family pyrin domain containing 3 (NLRP3) is known to be activated by mitochondrial ROS [16] and therefore it was selected to study the influence of mitochondrial oxidative stress. Fibroblast growth factor-23 (FGF23), a bone-derived hormone, although not directly linked to oxidative stress, is believed to influence systemic phosphate homeostasis, vitamin D metabolism, and alpha klotho expression [18], all factors which play roles in the oxidative stress balance. These genes were thus selected to provide an exhaustive spectrum of basic conditions.

## 2. Materials and Methods

### 2.1. Study Population and Selection Criteria

Ethical clearance was obtained from the Ethics Review Committee of the Postgraduate Institute of Science, University of Peradeniya. Informed consent for the study was obtained from each subject prior to blood collection. The samples were collected in different batches from June 2013 to January 2015.

**Figure 1:**
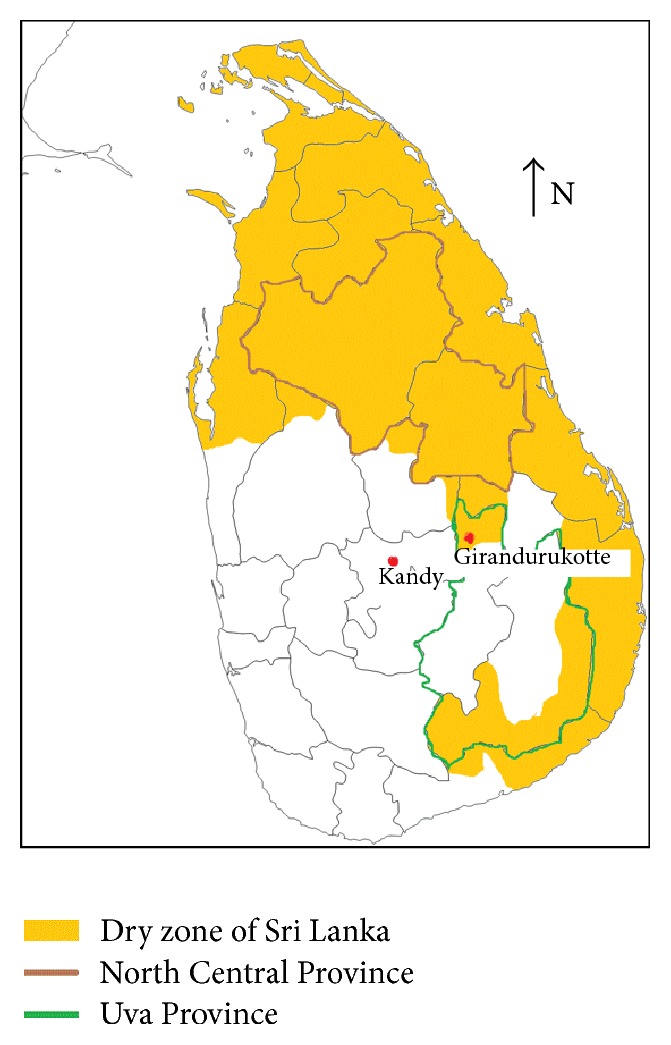
Map of Sri Lanka, highlighting dry zones of the country and study locations.

The study population was recruited in three study groups: CKDu, CKD, and healthy groups from Girandurukotte (GHI), which is a high prevalent area for CKDu located in the dry zone of Sri Lanka. The CKD and CKDu patients were recruited from the District Hospital, Girandurukotte. A control group of healthy individuals was selected from the Kandy District (KHI), which is not an endemic area of CKDu (Figure 1). The two healthy groups GHI and KHI were apparently healthy at the time of recruitment and did not show symptoms of any disease, did not have any past medical history, and were not previously diagnosed with any chronic diseases or infection. All study subjects belonged to the same race and ethnicity and only difference was on their place of residence.

The diagnosis of patients was carried out by the consulting nephrologist at the hospital and CKDu was diagnosed based on criteria set by the Ministry of Health, Sri Lanka; no past history of diabetes mellitus, chronic or severe hypertension, snake bite, glomerulonephritis, or urological diseases; normal HBA1C (<6.5%), blood pressure < 160/100 mmHg untreated, or <140/90 mmHg on up to two antihypertensive medications.

### 2.2. Inclusion and Exclusion Criteria

Inclusion criteria for the study were patients diagnosed by the physician as CKDu (irrelevant of the stage) and CKD patients with etiologies of diabetes or severe hypertension. Other etiologies of CKD were excluded from the study to limit variation. The quality and quantity of the extracted RNA were also factors determining inclusion to the study. The integrity of RNA as seen on agarose gel electrophoresis had to have 28s rRNA band approximately twice as intense as the 18s rRNA band and absorbance A260/A280 ratio of above 1.8. The quantity had to be sufficient for the preparation of cDNA. Samples that did not fulfill the above criteria were excluded.

### 2.3. Specimen Collection and RNA Extraction

RNA was extracted from whole blood (1 mL) using TRIzol LS reagent (Invitrogen, USA) according to manufacturer instructions. The RNA was further purified using PAXgene spin columns (PreAnalytix, USA).

### 2.4. Reverse Transcription Quantitative PCR (RT-qPCR)

cDNA was prepared by reverse transcription of 1 *μ*g of total RNA using QuantiTect Reverse Transcription kit (Qiagen) as described in the manufacturer protocol and quantitative PCR was carried out using QuantiTect Probe PCR kit (Qiagen) on a Rotor-Gene Q PCR cycler (Qiagen). Hydrolysis probes labeled with 5′FAM fluorescent dye and 3′BHQ quencher, as well as SYBR green mediated fluorescent detection were used for qPCR. Details of primer/probe pairs used in the study are listed (Table 1).

### 2.5. Data Analysis

Fold changes were calculated using the comparative Ct method [19]. Normalization was carried out against two reference genes, B2M and GAPDH, and individual fold changes were calculated using geometric mean of KHI males as calibrators. The log_2_ normalized fold changes were used to calculate outliers. The median values were calculated and extreme values extending to either side of 150% of the interquartile range (IQR) were considered outliers and not used in further calculations. Geometric means of expression fold changes for each gene in between the groups were calculated. The significance of the fold changes was calculated using one-way ANOVA, followed by post hoc Tukey analysis of significant results. Significant results were considered as those with *p* values < 0.05.

## 3. Results

The population included for the study from the Girandurukotte area consisted of CKDu patients (*n* = 43), CKD patients (*n* = 14) having etiologies of either hypertension, diabetes or both, and healthy individuals (GHI; *n* = 9). Healthy population were volunteers from the Kandy district (KHI; *n* = 16).

It is notable that the majority of the CKD and CKDu patients were farmers whereas the controls were of other occupations. The study population consisted of more males in accordance with an association that has been found with gender and proteinuric-CKD patients in a Sri Lankan population [20]. Patient demographics have been presented in Table 2.

### 3.1. Statistical Analysis

Fold changes and statistical significance as per one-way ANOVA and post hoc Tukey HSD analysis are summarized in Table 2. Of the studied genes, one-way ANOVA showed significant difference in between the groups for the GCLC (F_3,70_ = 9.659, *p* = 0.000), GSTM1 (F_3,49_ = 2.917, *p* = 0.043), NLRP3 (F_3,51_ = 5.111, *p* = 0.004), and FGF23 (F_3,45_ = 7.460, *p* = 0.000) genes. The G6PD gene did not show significant difference within the groups (F_3,48_ = 1.839, *p* = 0.153). Post hoc Tukey analysis showed that the upregulation of the GCLC gene in the CKDu (3.16-fold), CKD (4.10-fold), and GHI (7.27-fold) groups when compared to the KHI group was significant at *p* < 0.01. Notable was the high upregulation of this gene (7.27 fold) in the GHI compared to KHI (*p* < 0.01) (Table 3).

The GSTM1 gene did not show Ct values in 11 out of 43 CKDu patients (25.6%), six out of 14 CKD patients (42.9%), and eight of sixteen (50%) KHI. The population of the Girandurukotte area who expressed the gene, however, showed increased expression of the gene, with CKDu patients showing 2.60-fold upregulation compared to KHI (*p* = 0.036) and GHI showing 2.40-fold upregulation compared to KHI (*p* = 0.177; Table 2).

Post hoc analysis of the NLRP3 gene showed 6.55-fold (*p* = 0.006) and 11.30-fold (*p* = 0.010) upregulation in the CKDu and CKD groups, respectively, whereas FGF23 showed a similar pattern with 21.91-fold (*p* = 0.003) and 134.97-fold (*p* = 0.001) upregulation in the CKDu and CKD groups.

**Figure 2:**
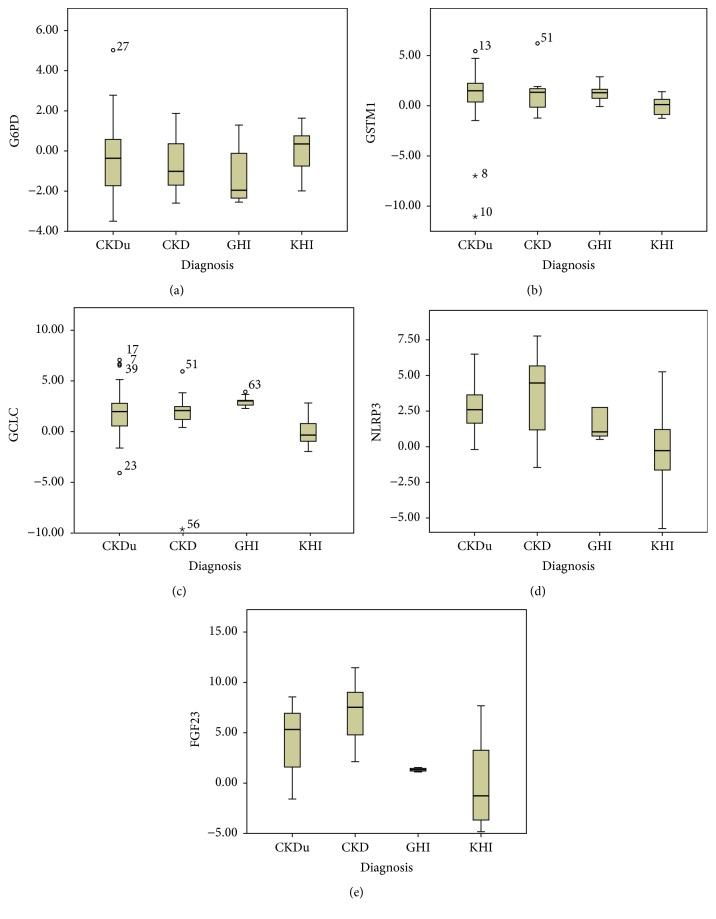
Box plots showing median values and range of log⁡_2_ normalized fold changes of (a) G6PD, (b) GSTM1, (c) GCLC, (d) NLRP3, and (e) FGF23 genes in the four groups: CKDu, CKD, GHI, and KHI. Outliers in each group are depicted by (∘) for values exceeding 150% IQR and (⋆) for values exceeding 200% IQR. Numbers alongside symbols indicate sample ID.

**Figure 3:**
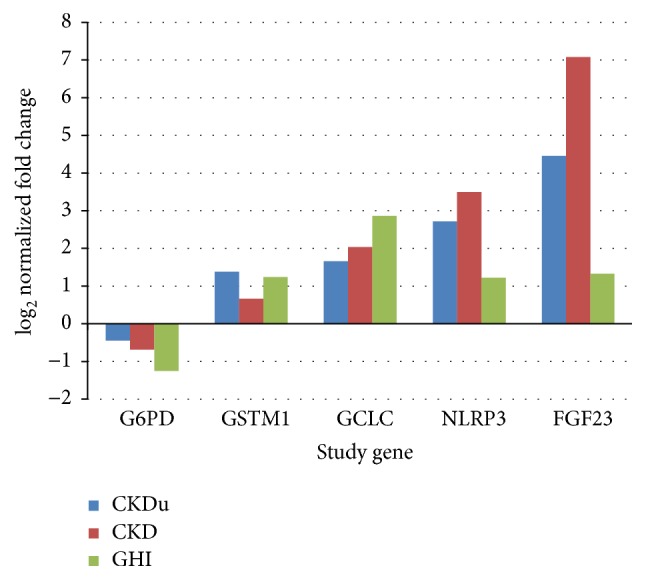
log⁡_2_ normalized fold change of the study genes in the four study groups.

Box and whisker plots depicting median values and outliers (150% IQR and 200% IQR) for each gene in each study group are presented (Figure 2). Average log_2_ normalized fold changes calculated following outlier removal are depicted graphically in Figure 3.

## 4. Discussion

Gene expression studies based on renal biopsies have made an important contribution to understanding the role of transcriptional regulation in renal disease [21]. Studying the expression patterns of genes in CKDu-endemic populations could give a better understanding of the influential factors of chronic kidney disease in this population. For this study, genes known to be functioning in the oxidative stress balance were selected, for their expressions to be studied in blood of populations of both a CKDu-endemic region as well as a nonendemic region. Genes not directly related to oxidative stress, but indicative of the sources of oxidative stress were also selected to be studied. As CKDu is believed to be a result of occupational and environmental conditions, it was hypothesized that the study of oxidative stress related genes would help better understand the influence of the environment in this disease.

The GCLC and GSTM1 genes were selected for the study based on their relation to the glutathione (GSH) protein. GSH functions in protecting the cells from toxic compounds, oxidative damage, and radiation and forms complexes with several metal ions, thus protecting cells from metal toxicity as well [22]. It is used as a cosubstrate in the detoxification of electrophilic xenobiotics and peroxides [23]. GSH is also considered the first line of defense in metal toxicity before even metallothionein [22]. The expression of the GCLC gene was seen to be upregulated more than 3.16-fold in all three study groups from the Girandurukotte region; the CKDu, CKD, and GHI when compared to the KHI (*p* < 0.01). GCLC catalyzes the rate-limiting step in the production of GSH and synthesis of GSH is not possible in the absence of GCLC. Its activity is highest in the liver, followed by the lungs and kidneys, and the steady state levels of the enzyme vary in different tissue types and stages of development [23]. The GSH effect depends on the balance of depletion, regeneration, and synthesis of the molecule [24] and when the GSH is depleted, the GSH synthesizing systems start making more GSH [25]. The increased production of this enzyme in blood of the Girandurukotte study groups indicates increased production of GSH to counter oxidative stress. The GHI group had the highest expression of this gene at 7.27-fold upregulation and this is possibly a reason for their being healthy and not showing symptoms of the disease.

The GSTM1 gene belongs to a group of enzymes that is responsible for the conjugation of GSH to electrophilic xenobiotics thereby playing a key role in the biotransformation and detoxification of xenobiotics and endogenous substances [14]. It was seen in our study population that GSTM1 gene did not show Ct values in 11 out of 43 CKDu patients (25.6%), six out of 14 CKD patients (42.9%), and eight out of sixteen (50%) of the KHI. All nine individuals of the GHI group had expression of the gene. The CKDu patients who expressed the gene, however, showed increased expression with 2.60-fold upregulation (*p* < 0.01) compared to KHI. It can be inferred that absence of the gene in a risk environment may be further aggravating disease progression.

The lack of the GSTM1 gene expression in 25.6% of the CKDu patients and 42.9% of the CKD patients and 50% of the KHI population indicates the possibility of a null mutation in this ethnicity. The GSTM1 null mutation is a common polymorphism and has also been associated with the end-stage renal disease of unknown etiology in Mexican patients [14]. The GSTM1 null polymorphism individuals were also found to be more vulnerable to organochlorine pesticides leading to the development of CKDu in a study carried out in an Indian population [15]. The lack of expression of the gene observed in 25.6% of our CKDu patients and 42.9% of the CKD patients indicates that they are vulnerable and susceptible to oxidative stressors. While 50% of the KHI also did not express the gene, their good health indicates that they are not being exposed to the same level of oxidative stress. The groups who had expression of the gene, including the GHI group, although not statistically significant, had upregulation of the gene. This again indicates regulation taking place to counter oxidative stress. The individuals of the KHI group who expressed the GSTM1 gene had lower expression compared to the study groups from Girandurukotte.

The transcription of genes encoding the detoxifying and antioxidant enzymes such as the GSTs and GLCs are stimulated in response to cytoplasmic oxidative stress [26]. Among other oxidative stressors, the expression of GCLC gene has been associated with countering metal toxicity, due to its metal binding sites and has especially been correlated with cadmium toxicity [24] which has been a major contender as the causative agent of CKDu.

The alteration of expressions of the GSH related genes in the populations of the Girandurukotte region, both healthy and diseased, could be further interpreted based on the functions of GSH and the current hypotheses of CKDu. The upregulation of the GSH related genes in these groups indicates that there is/are certain environmental factor/s causing depletion of GSH, thereby inducing expression of genes to counteract it. GSH acts as a metal chelating agent and oxygen radical scavenger [24]. Redox-inactive metals including lead, mercury, cadmium, and arsenic indirectly cause oxidative stress by depletion of the cells' major sulfhydryl reserves [27]. The GSH tripeptide molecule makes up more than 90% of the total nonprotein sulfur and the redox-inactive metals interact with the GSH metabolism as an essential part of its toxic response [25].

Another gene that we studied that is related to oxidative stress and plays a role in protection against reactive oxygen species is the gene responsible for the production of the glucose-6-phosphate dehydrogenase (G6PD) enzyme. G6PD deficiency is a very common enzymopathy in the world as well as in CKDu prevalent areas of Sri Lanka [13]. The study by Jayasekara et al. found 20% of the CKDu cases to have some severity of G6PD deficiency whereas only 2% of the healthy controls had the deficiency [13]. This enzyme is the first rate-limiting enzyme in the pentose phosphate pathway and is responsible for the production of NADPH which is needed in the glutathione reduction process [28]. However, the differential expressions seen in our study groups were not statistically significant on the one-way ANOVA.

Our finding based on the GSH related gene expression indicates comparatively less oxidative stress in the KHI and the possibility that the oxidative stress factor/s is/are of an environmental source. Many studies have based their hypotheses on the fact that the drinking water sources differ in the affected communities. The Kandy population consumes water supplied via pipelines after water treatment, whereas the Girandurukotte population had mainly groundwater sources. Studies have analyzed the amount of trace and ultra trace elements in drinking water and have found moderate to high levels of fluoride in affected regions [29]. Wasana et al. [30] found strong relation between synergistic effects of fluorine, cadmium, and hardness of drinking water and prevalence of CKDu. Dharma-wardana et al. [31] hypothesize that CKDu in the NCP is linked with fertilizer runoff into the river systems from agricultural activity in the central hills which increases the ionicity of the drinking water which has potential to further damage the protein structures in the kidney. Exposure to pesticides has also been implicated in the induction of oxidative stress and they can disturb the oxidative balance through either direct or indirect pathways including thiol oxidation [32]. Urine cadmium and arsenic concentrations in individuals have been in some studies found to be at levels known to cause kidney damage [33]. The role of GSH in counteracting arsenic toxicity has also been documented. Exogenous GSH administered to rats has also helped to reduce arsenic species in liver and to stop the decrease of blood and hepatic glutathione and total antioxidant capacity [34]. Algal toxins are another hypothesized cause for CKDu as cyanotoxin producing algae have been found in drinking water of certain CKDu-affected regions [35]. GSTM3, another member of the GST family, was one of the phase II enzymes found to be upregulated in hepatoma HepG2 cells in the detoxification response to a cyanotoxin, cylindrospermopsin [36].

The sources of the rice, which is the main staple of the Sri Lankan people, and the drinking water were the main differences between the two groups and therefore the oxidative stress could also be related to this factor. The Girandurukotte population had rice from their own production whereas rice purchased in Kandy comes from different parts of the country. The climate between the two areas is also different; Girandurukotte is of the dry zone and has temperatures averaging 28.1°C and 22 mm rainfall in the warmest and driest periods of May and June, respectively [37], whereas Kandy being of the wet zone and having temperatures averaging 25.9°C and 100 mm rainfall in the warmest and driest periods of April and February, respectively [38]. The people of Girandurukotte, especially those involved in farming, are exposed to the high temperatures and have a threat of dehydration [39]. Consumption of contaminated water in a dehydrated state further concentrates the effect of the possible toxins.

ROS are however not only from external sources but also generated within cells by aerobic respiration as well as by anabolic and catabolic reactions taking place within the cells [40]. The influence of metabolic dysregulation might also be a major contributor to oxidative stress in these patients. The NLRP3 gene was selected due to its activity being dependent on mitochondrial ROS production. The mitochondria are the largest producer of the ROS within cells due to the electron leak from the electron transport chain [40]. The increased oxidative stress in chronic kidney disease is in part attributed to the dysfunctional mitochondria which cause an increased electron leakage from the respiratory chain during oxidative phosphorylation with a consequent generation of ROS [16]. We observed an upregulation of the NLRP3 gene in both the CKDu and CKD patients with fold changes of 6.55 and 11.30, respectively (*p* < 0.01). ROS generation by mitochondria plays an important role in the NLRP3 inflammasome where blocking the ROS generation by mitochondria was seen to even stop the NLRP3 inflammasome activation [41]. Further hyperuricemia, which occurs with deteriorating renal function [42], induces oxidative stress as a primary deleterious effect [43]. Chronic hyperuricemia promotes mitochondrial dysfunction and decreased ATP content which results in renal oxidative stress. The mitochondrial dysfunction caused by hyperuricemia could further increase electron leakage and increase ROS production. The fold changes observed suggest more involvement of these metabolic pathways and cellular influence of oxidative stress in the CKD patients.

FGF23, a bone-derived hormone, is believed to influence systemic phosphate homeostasis, vitamin D metabolism, and alpha klotho expression [18, 44, 45] and is therefore indirectly linked to ageing and oxidative stress generated by dysregulation of these metabolic processes. It is believed to have a protective response to prevent excess phosphate retention, which leads to a reduction in vitamin D [18, 46], which could further worsen oxidative stress conditions [18]. Serum FGF23 levels are seen to rise exponentially as renal function declines [44]. A significant level of upregulation of the FGF23 gene was seen in our CKD and CKDu populations of 134.97-fold and 21.94-fold, respectively (*p* < 0.01). FGF23 levels have even been seen to reach 10,000-fold higher in end-stage renal disease when compared to healthy individuals [47]; however, the comparatively lower fold changes in our study could be due to the fact that the gene expression levels were analyzed in whole blood which is not the primary tissue of production of the FGF23. The increase seen is however sufficient to observe the regulation of this gene in relation to CKD and CKDu. Similar to the NLRP3 gene, CKD population showed higher expression of the FGF23 gene than the CKDu population, which again points towards differing pathophysiological conditions and sources of oxidative stress in the two disease groups.

The stage of disease and administered medication will further have an impact on the expression of the said genes. The patients in our study, both CKD and CKDu, are treated conservatively to manage symptoms and prescribed supplemental iron, folic acid, and calcium, while patients identified with diabetes and cholesterol are further given respective medications while monitoring blood sugar and cholesterol levels. Calcium supplementation is an influencing factor in FGF23 regulation [48] and this could therefore be influencing results observed of the gene, but the effect should become nullified when comparing the CKD and CKDu groups who are both given the supplementation.

The cellular source of oxidative stress is playing a bigger role in CKD patients, whereas there is a possible environmental source of oxidative stress which is not present in the Kandy region that is causing the upregulation of the GSH related genes in the GHI group. The GHI population, although showing increase of GCLC expression, showed relatively slight increase of NLRP3 and FGF23 genes which was not of statistical significance. This shows the lack of cellular mediated oxidative stress in this group but the presence of other factors that could influence GCLC upregulation. Several extracellular stimuli cause ROS production, including growth factors, inflammatory cytokines, ionizing radiation, UV, chemical oxidants, chemotherapeutics, hyperoxia, toxins, and transition metals [40]. The oxidative stressors could be any of the risk factors hypothesized in the literature, including but not limited to metal toxicity, fluoride toxicity, agrochemical toxicity, and algal toxicity, which have been correlated with disease prevalent areas and linked to water sources.

## 5. Limitations of Study

The physiological and pathological conditions of an individual is a summed effect of many different genes and influences of external and internal factors and therefore cannot simply be linked to the few genes analyzed in this study. However, GSH plays a significant role in the antioxidant protective system of the body, and as GCLC and GSTM1 genes play significant roles in the production and function of GSH, their patterns can be indicative of the physiological condition of an individual. The FGF23 gene expression was studied in blood tissue, which is not its primary tissue of production, but the expression seen is sufficient to infer possible influences of the gene in the study groups. Other limitations included small study population and also age difference in between the healthy groups and the disease groups. The two healthy groups were however of the same age range and therefore does not affect the conclusion of this study. The groups could be further divided stage-wise in a larger sample size to observe changes of the genes with disease progression.

## 6. Conclusion

The most significant finding of the study was the higher upregulation of the GCLC gene in the GHI group compared to the other study groups. The GSTM1 null mutation in the Girandurukotte population resulted in disease, but this was not so in the Kandy population. Patients showed effects of mitochondrial source of oxidative stress as well as metabolic dysregulation as seen with NLRP3 and FGF23 upregulation, more in CKD than in CKDu, pointing at different triggers of oxidative stress in the two disease subtypes. GHI did not show significant upregulation of the NLRP3 or FGF23 genes; therefore, indicating that the GCLC upregulation is externally stimulated. From this pilot study, we can therefore conclude that there is an environmental source of oxidative stress in the Girandurukotte region which is not present in the Kandy region. It can be inferred that GSH plays a protective role in keeping individuals of the Girandurukotte area healthy and the lack thereof promotes disease. Studies with a wider array of genes and stage-wise classification are currently being carried out to further understand molecular mechanisms of the disease and to identify causative etiology.

## Figures and Tables

**Table 1:** Primer and probe sequences used for the study.

Name	Accession number	Amplicon length (base pairs)	Oligonucleotide sequences 5′–3′ (F: forward, R: reverse, P: probe)	References
GCLC	NM_001498.3	149	F: ACAAGGACGTTCTCAAGTG	Self-designed
			R: AGGATGGTTTGGGTTTGTC	
			P: CCTGTCTGGGGAGAAAGTTCTTGAAACTCT	

GSTM1	NM_000561.3	121	F: GCATGATCTGCTACAATCCA	Self-designed
			R: TTGTTTCCTGCAAACCAT	
			P: CCTGAAAAGCTAAAGCTCTACTCAGAG	

G6PD	NM_000402.4	76	F: TGCCCCCGACCGTCTAC	*RTPrimer DB*
			R: ATGCGGTTCCAGCCTATCTG	
			P: ACTCGTGAATGTTCTTGGTGACGGCC	

NLRP3	NM_004895.4	225	F: CTCCTTTACGCCAGGGTGAG	Self-designed
			R: AGATAGCGGGAATGATGATATGAG	

FGF23	NM_020638.2	120	F: AATCTCAGCACCAGCCACTC	Self-designed
			R: GGAGGCATTGGGATAGGCTC	

B2M	NM_004048.2	98	F: TGCCGTGTGAACCATGTGA	Koop et al., 2003 [17]
			R: CCAAATGCGGCATCTTCAA	
			P: TGATGCTGCTTACATGTCTCGATCCCACT	

GAPDH	NM_001289745.1	151	F: TGACCTCAACTACATGGTTTA	Self-designed
			R: GCCCCACTTGATTTTGGA	
			P: CCATGGCACCGTCAAGGCTGA	

**Table 2:** Demographics of study population.

	CKDu (*n* = 43)	CKD (*n* = 14)	GHI (*n* = 9)	KHI (*n* = 16)
Gender	Males = 37	Males = 13	Males = 8	Males = 15
	Females = 6	Females = 1	Females = 1	Females = 1
Age (years)	52.0 ± 9.2	58.2 ± 10.7	40.0 ± 11.3	34 ± 8.1
Occupation	Farmers = 38	Farmers = 12	Farmers = 0	Farmers = 0
	Nonfarmers = 5	Nonfarmers = 2	Nonfarmers = 9	Nonfarmers = 16
Area of residence	Girandurukotte	Girandurukotte	Girandurukotte	Kandy

**Table 3:** Fold change in expression of genes in the study groups compared to KHI and their statistical significance as per one-way ANOVA followed by post hoc Tukey analysis.

Gene	Fold change compared to KHI group	Statistical significance, *p*
G6PD^*∗*^	CKDu, 0.73	0.703
*N* = 75	CKD, 0.62	0.562
*O* = 1	GH, 0.42	0.137

GSTM1	CKDu, 2.60	0.036
*N* = 53	CKD, 1.58	0.729
*NE* = 25	GH, 2.37	0.177
*O* = 4		

GCLC	CKDu, 3.16	0.001
*N* = 74	CKD, 4.10	0.002
*NE* = 1	GH, 7.27	0.000
*O* = 7		

NLRP3^*∗*^	CKDu, 6.55	0.006
*N* = 54	CKD, 11.30	0.010
*NE* = 3	GH, 2.38	0.553

FGF23^*∗*^	CKDu, 21.94	0.003
*N* = 49	CKD, 134.97	0.001
*NE* = 6	GH, 2.52	0.904

*N*: number included for one-way ANOVA

*O*: outliers

*NE*: number of samples not expressing gene

^*∗*^total sample less than 82 due to insufficient sample amount.
